# Supplementary material for: A chromatinized origin reduces the mobility of ORC and MCM through interactions and spatial constraint
Source: Nat Commun. 2023 Oct 23;14:6735. doi: 10.1038/s41467-023-42524-8 (PMC10593741; doi:10.1038/s41467-023-42524-8)
Supplement: Supplementary file 1 — Supplementary information [file 41467_2023_42524_MOESM1_ESM.pdf]

Supplementary figures for:

A chromatinized origin reduces the mobility of ORC and MCM through interactions and spatial constraint

Humberto Sánchez[1], Zhaowei Liu[1], Edo van Veen[1], Theo van Laar[1], John F. X. Diffley[2], and Nynke H. Dekker[1*]

Affiliations:

[1]Department of Bionanoscience, Kavli Institute of Nanoscience, Delft University of Technology, Delft, The Netherlands.
[2] Chromosome Replication Laboratory, Francis Crick Institute, London, UK.

*Correspondence: n.h.dekker@tudelft.nl

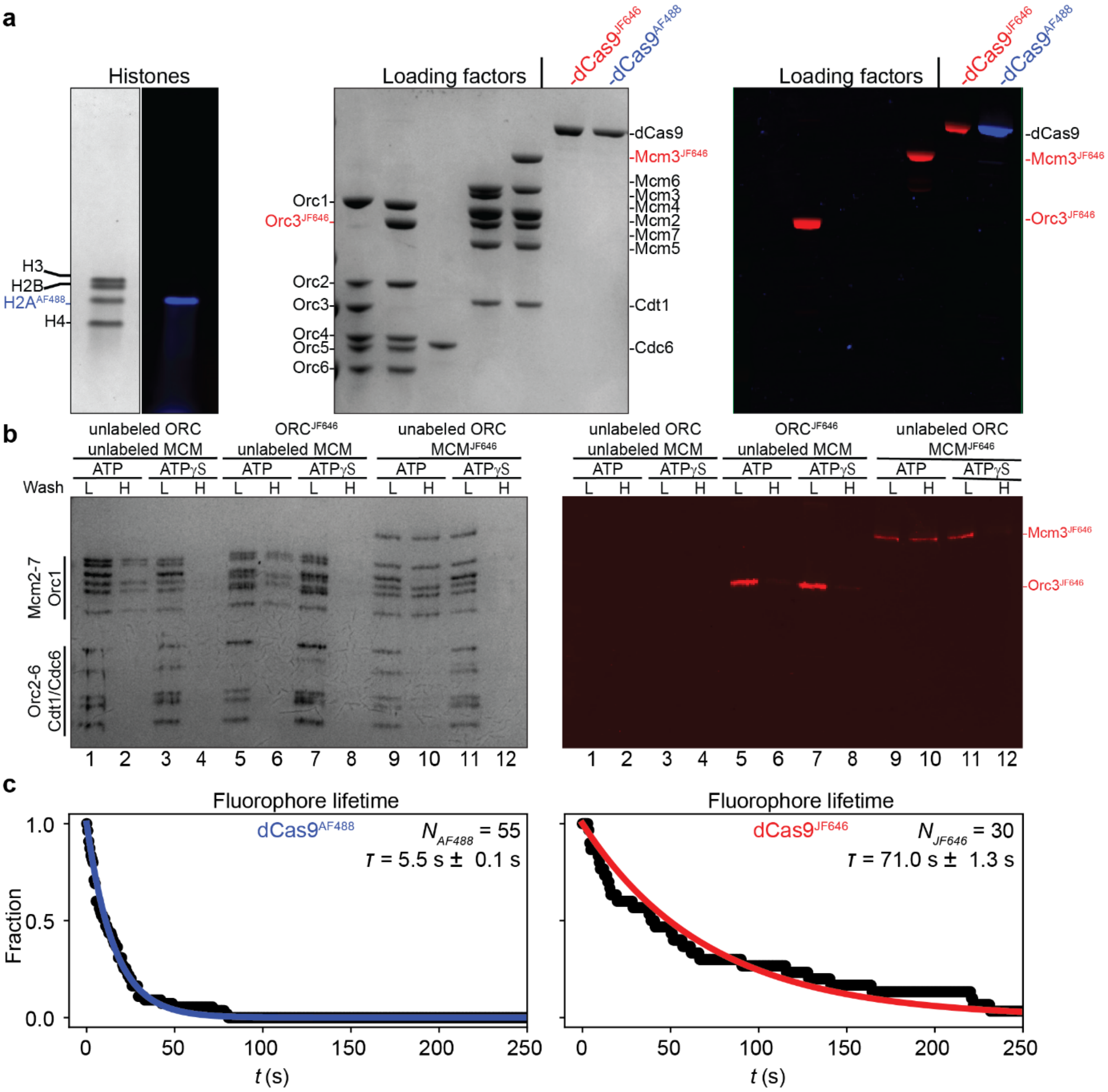

Supplementary Figure 1.1. Protein purification and ensemble characterization. (a) Scans from the SDS-PAGE gels after Coomassie staining (in black and white) and after fluorescence imaging (in color) of the yeast histone octamer complex (containing labeled $H2A^{AF488}$), loading factors (ORC complex, ORC^{JF646} complex including a HaloTag on the Orc3 subunit labeled with a single JF646 dye, Cdc6, MCM/Cdt1 complex, MCM^{JF646}/Cdt1 complex including a HaloTag on the Mcm3 subunit labeled with a single JF646 dye), and fluorescence standards dCas9 labeled with Halo-JF646 and Halo-AF488, respectively. **(b)** Loading assay performed on bead-tethered DNA in bulk using unlabeled proteins, or labeled ORC complex (ORC^{JF646}) with unlabeled MCM/Cdt1, or unlabeled ORC complex with labeled MCM^{JF646}/Cdt1, as indicated. Following a

reaction performed in the presence of either ATP or ATPγS, either a low (L) or a high-salt (H) wash is performed. DNA is removed from the beads by MNase treatment, and the presence of DNA-bound proteins is examined using SDS-PAGE followed by fluorescence scanning (color image) and silver staining (b/w). Mcm2-7 proteins are retained on the DNA following a high-salt wash only if the loading reaction was performed in the presence of ATP. This confirms recruitment of Mcm2-7 in the presence of ATPγS, but loading of Mcm2-7 only in the presence of ATP (lanes 2, 6, and 10). **(c)** Single-molecule lifetime analysis of the fluorophores used in this study obtained at an acquisition frequency of one frame per 0.6 s. (left panel) Survival probability (fraction of fluorophores still visible at a given time following initial illumination) of single AF488 dyes in dCas9^{AF488} (N_{AF488}), which yields their timescale of photobleaching. (right panel) Survival probability of single JF646 dyes in dCas9^{JF646} (N_{JF646}), which yields their timescale of photobleaching. The black dots represent the data, and the solid curves are exponential decay fits to the data. Data are presented as mean values ± standard deviation, computed from the covariance of the optimal parameters found by the least squares fit. Source data are provided as a Source Data file.

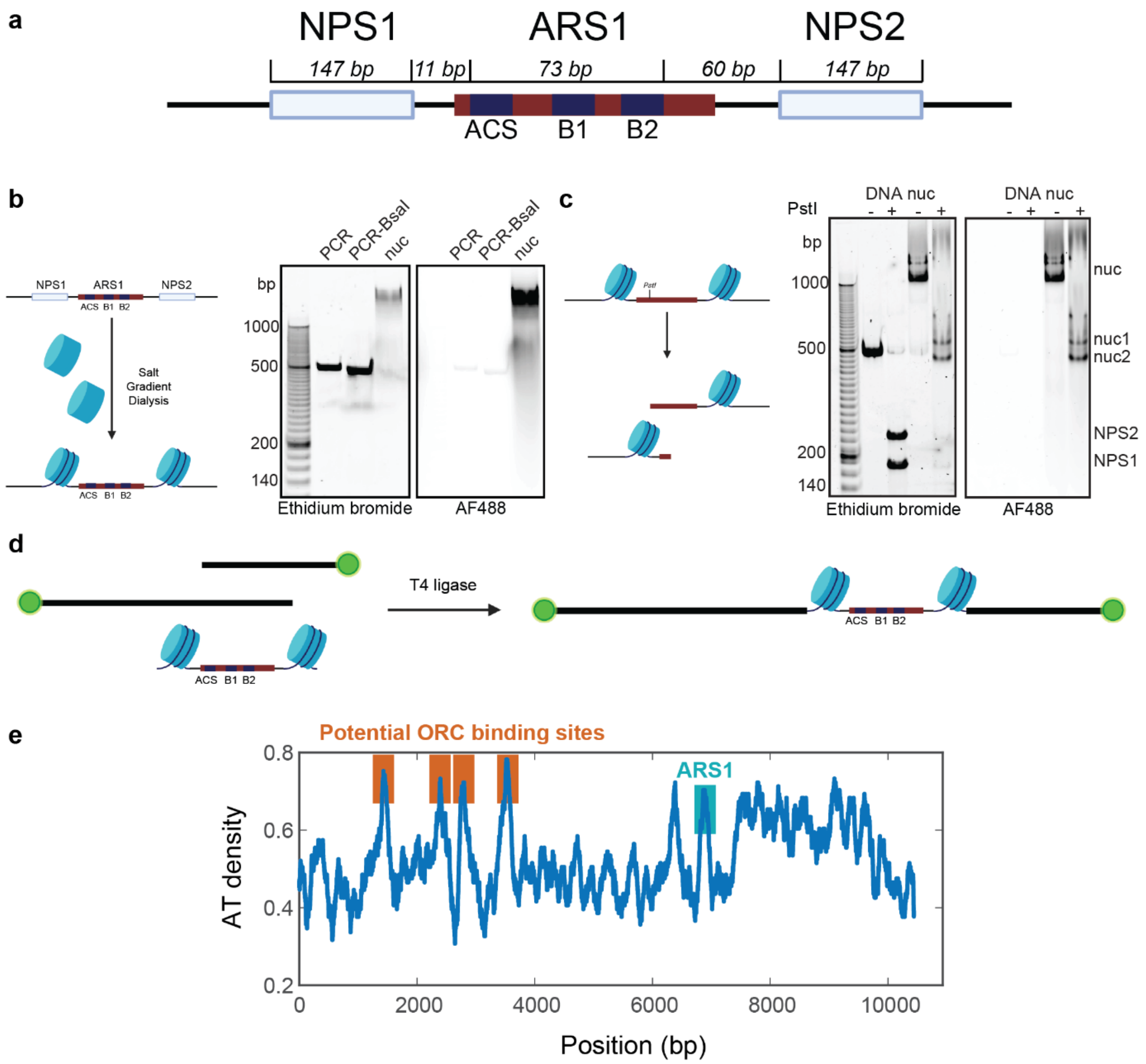

Supplementary Figure 1.2. Establishment of a chromatinized origin on a 10.4 kbp DNA. (a) Detailed map (not to scale) of the region of the DNA molecule used in this study that contains the ARS1 origin of replication (ACS, B1, and B2 elements highlighted in dark blue boxes) flanked by two NPS sites, with all lengths indicated in base pairs. **(b)** (left panel) Schematic of a PCR fragment that contains the ARS1 origin of replication flanked by two NPS sites used for nucleosome assembly by salt gradient dialysis. (right panel) Scans from a native PAGE gel after ethidium bromide staining (left) and after fluorescence imaging (right) of the PCR fragment, PCR fragment with cohesive ends, and PCR fragment with cohesive ends with assembled nucleosomes prior to ligation. **(c)** (left panel) Schematic of the restriction enzyme accessibility

assay performed to determine the degree of NPS site occupancy. (right panel) Scans from a native PAGE gel after ethidium bromide staining (left) and after fluorescence imaging (right) of the PCR with cohesive ends (DNA) without (-) or with (+) PstI, and nucleosomes assembled on the DNA (nuc) without (-) or with (+) PstI. **(d)** Schematic of the ligation protocol using T4 ligase to generate the final 10.4 kbp DNA from the assembled nucleosomes and the biotinylated 'arms'. **(e)** Nucleotide density analysis along the 10.4 kbp DNA construct in sliding windows of 100 bp. ORC near-cognate binding sites and the ARS1 site position are indicated in the plot. Source data are provided as a Source Data file. Created with BioRender.com.

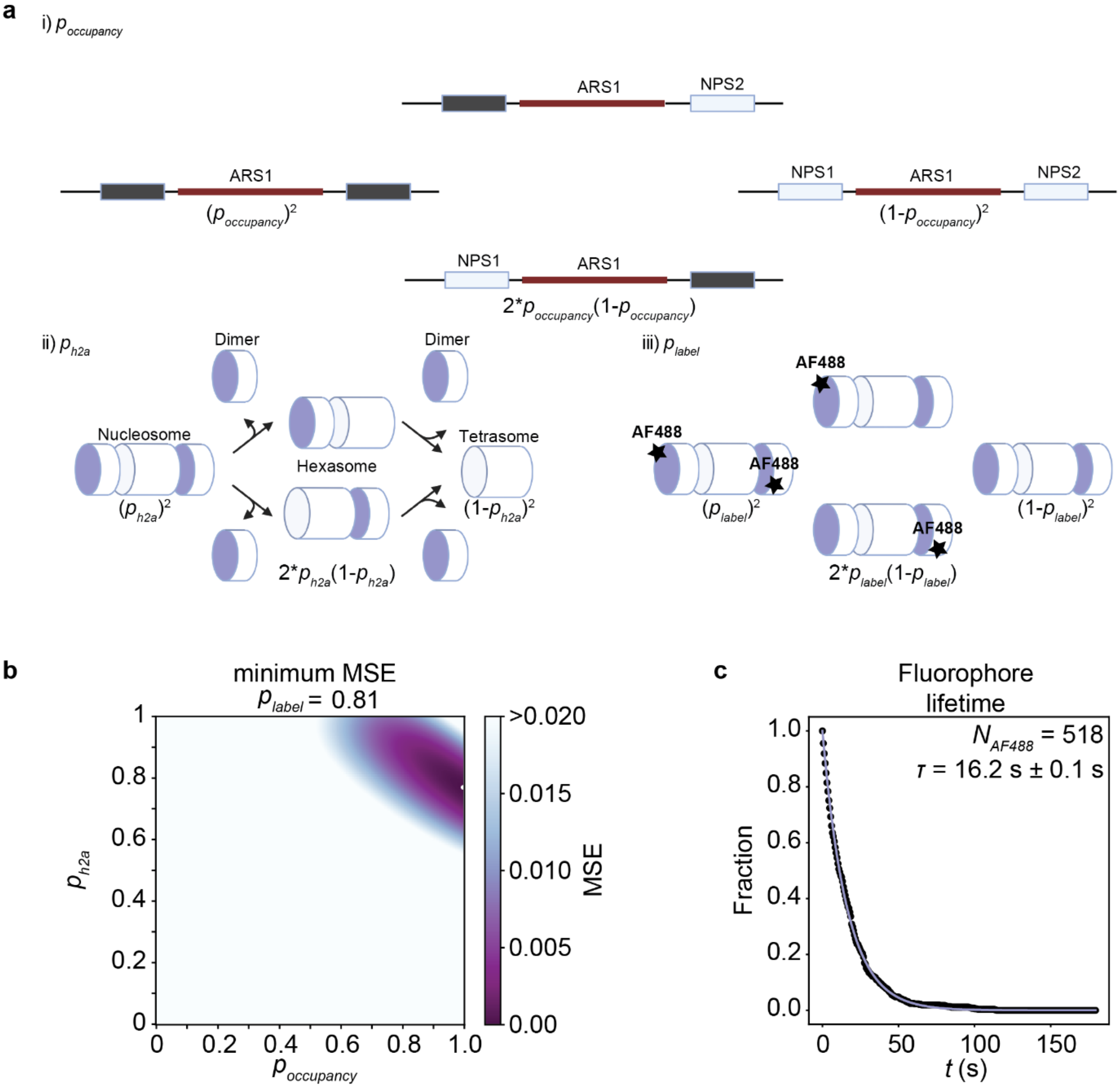

Supplementary Figure 1.3. Model for extracting biophysical parameters from the H2A^{AF488} stoichiometry distributions. (a) Three factors influence the observability of H2A^{AF488} at the two NPS sites in our experiments: (i) Both, one, or none of the NPS sites can be occupied ($p_{occupancy}$, free parameter); dark box: occupied; light box: not occupied. (ii) An NPS site can be occupied by a tetrasome, hexasome, or nucleosome, depending on whether 0, 1, or 2 H2A^{AF488}-H2B dimers are bound to a tetrasome, where by the probability of dimer binding is given by p_{h2a} (free parameter). (iii) The dimers will be visible or not depending on the labeling efficiency of H2A^{AF488} (experimentally determined and input into the model as p_{label}). See Methods for a complete mathematical description. Created with BioRender.com. **(b)**

Landscape of the mean squared error (MSE) between fit and experiment calculated for different values of p_{h2a} and $p_{occupancy}$ at a fixed value of p_{label} = 0.81. The white dot indicates the optimal solution with the lowest error (p_{h2a} = 0.77, $p_{occupancy}$ = 1.00). **(c)** Single-molecule lifetime analysis of H2A. Survival probability of all (N_{AF488} = 518) single AF488 dyes in $H2A^{AF488}$ on DNA containing an origin, as deduced from the diffraction-limited blue spots collected from 248 distinct DNA molecules at an acquisition frequency of one frame per 0.6 s. Data are presented as mean values ± standard deviation, computed from the covariance of the optimal parameters found by the least squares fit. Source data are provided as a Source Data file.

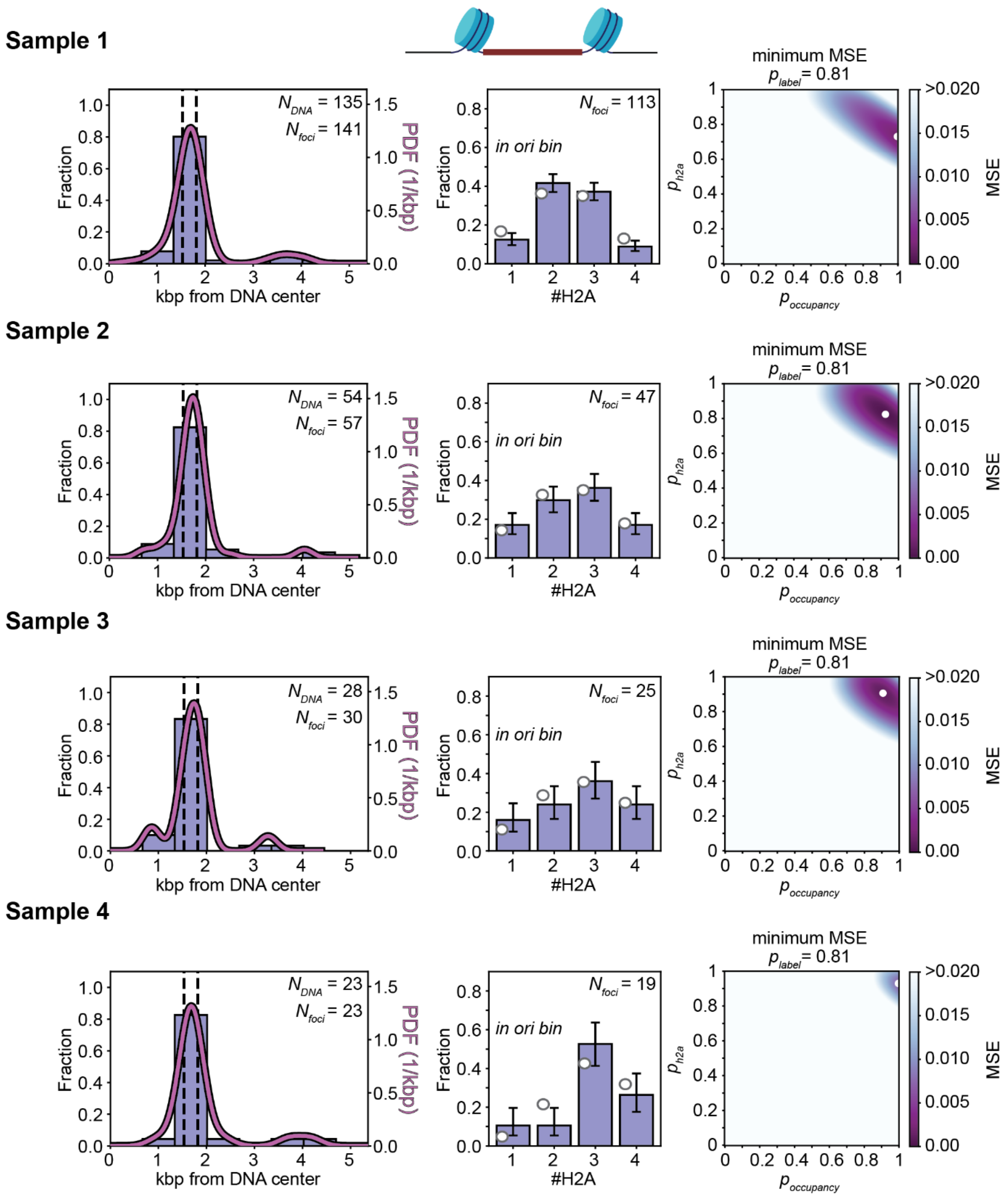

Supplementary Figure 1.4. Employed sample preparations of a labeled chromatinized origin within 10.4 kbp DNA. (left panels) Spatial distribution of H2A^{AF488}on DNA containing an ARS1 origin as described in **Figure 1b**, acquired immediately after introduction into the flow cell and deduced from the blue diffraction-limited spots (N_{foci}) collected from a number of DNA molecules indicated by N_{DNA}. Dashed lines indicate the location of the NPS sites, and the solid

curve indicates the kernel density estimation of the data (PDF: probability density function). (center panels) Stoichiometry distribution of $H2A^{AF488}$ in the bin containing the chromatinized origin. Data are presented as mean values ± one-sigma Wilson confidence intervals. Filled white circles indicated at left designate the fitted values based on the model described in the Methods and in **Supplementary Figure 1.3**. (right panels) Landscapes of the mean squared error (MSE) between fit and experiment, calculated for different values of p_{h2a} and $p_{occupancy}$ at a fixed value of p_{label} = 0.81. The white dots indicate the optimal solutions with the lowest error. Source data are provided as a Source Data file. Created with BioRender.com.

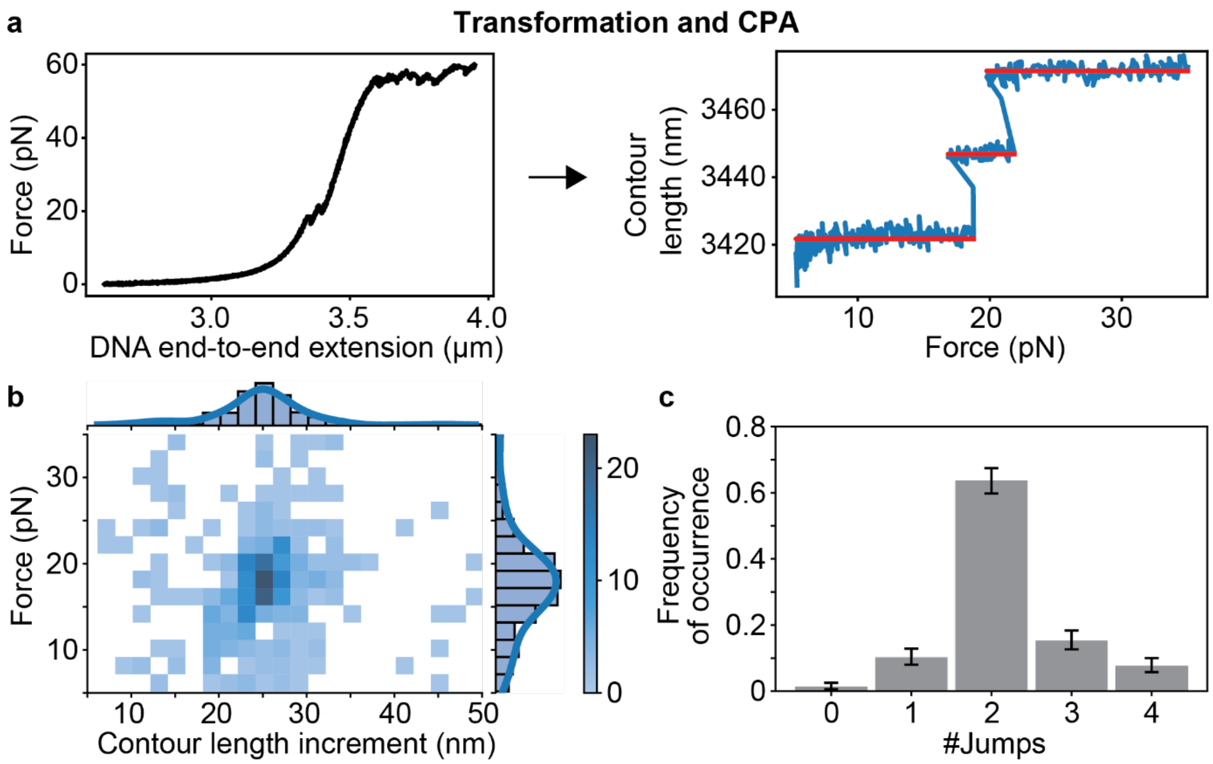

Supplementary Figure 1.5. Force spectroscopy of 10.4 kbp DNA containing a chromatinized ARS1 origin. (a) (left panel) Representative force-extension curve for a typical DNA containing a chromatinized ARS1 origin. Unwrapping of the DNA from the nucleosomes (formed with $H2A^{AF488}$) results in observable increases in the end-to-end extension of the DNA and concomitant decreases in the force[23]. The extent of DNA unwrapped from the nucleosomes is assessed by transforming the data into a contour length-force plot and performing change point analysis (CPA) to detect the plateaus. **(b)** Heatmap of the distribution of force ($\langle F_{unwrap} \rangle$ = 17.6 pN ± 5.8 pN, mean ± SD) and contour length increments ($\langle \Delta L_c \rangle$ = 24.8 nm ± 5.9 nm, mean ± SD) found by CPA in 157 molecules. **(c)** Distribution of the number of contour length increments per chromatinized DNA molecule. Data are presented as mean values ± one-sigma Wilson confidence intervals.

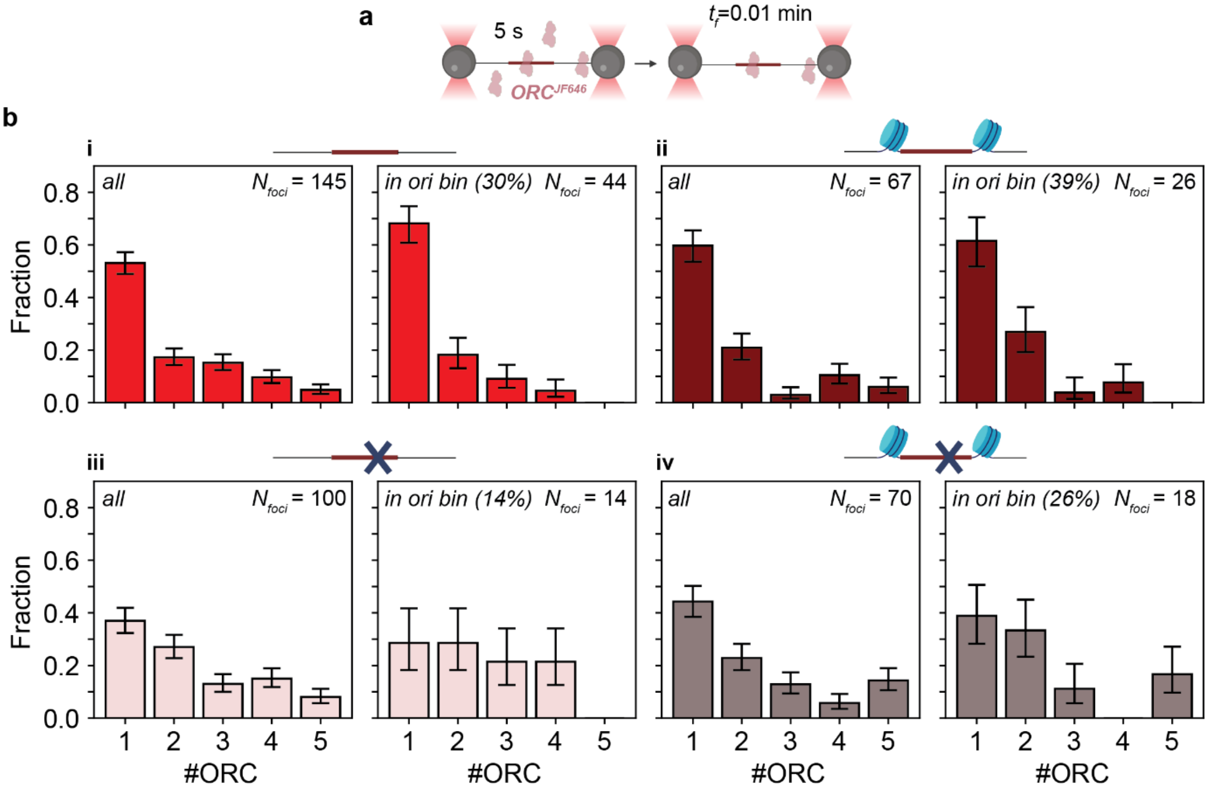

Supplementary Figure 2. Stoichiometry of rapidly bound ORC^{JF646} on 10.4 kbp DNA containing an ARS1 (or mutated) origin (chromatinized or not). (a) Tethered DNA molecules (chromatinized or not, as indicated) are introduced into the protein channel (Ch4) for incubation with ORC^{JF646} and Cdc6 for 5 s in the presence of ATP, and then moved into the buffer channel (Ch3) for confocal scanning at an acquisition frequency of one frame every 0.01 min (t_f = 0.01 min) (see also **Figure 2**). **(b)** Stoichiometry of ORC^{JF646}- foci (N_{foci}) pertaining to data shown in **Figure 2.** (left panels) Stoichiometry distributions of ORC^{JF646} over the entire DNA molecule and (right panels) stoichiometry distribution of ORC^{JF646} in the bin containing (i) the non-chromatinized ARS1 origin; (ii) the chromatinized ARS1 origin; (iii) the non-chromatinized mutated origin; and (iv) the chromatinized mutated origin, as also indicated by the schematics. Data are presented as mean values ± one-sigma Wilson confidence intervals. Created with BioRender.com.

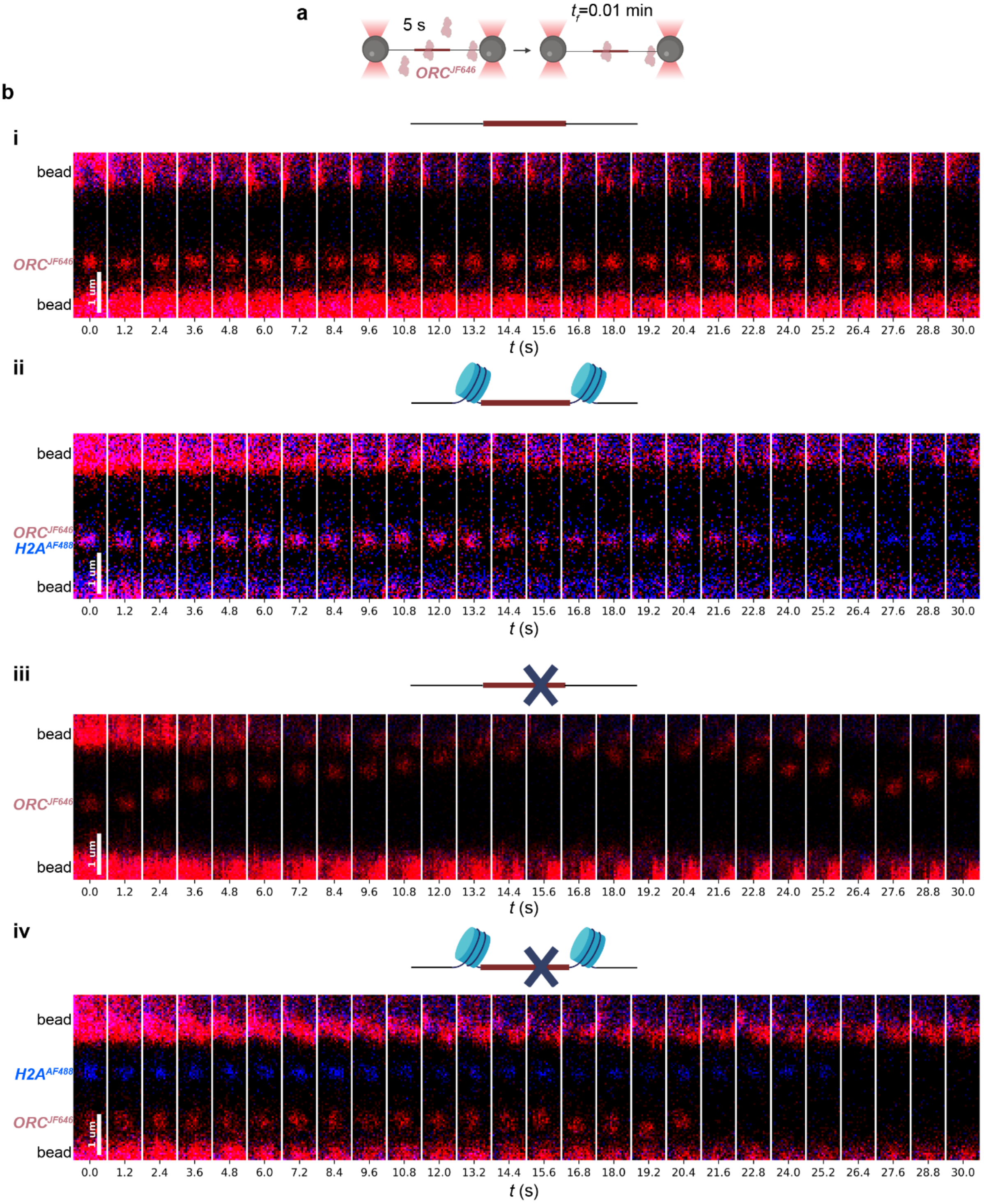

Supplementary Figure 3.1. Representative kymographs for rapidly bound ORC^{JF646} on 10.4 kbp DNA containing an ARS1 (or mutated) origin (chromatinized or not). (a) Tethered DNA molecules (chromatinized or not, as indicated) are introduced into the protein channel (Ch4) for incubation with ORC^{JF646} and Cdc6 for 5 s in the presence of ATP, and then moved into the buffer channel (Ch3) for confocal scanning at an acquisition frequency of one frame every

0.01 min (t_f = 0.01 min) (see **Figure 1b** for schematic of the flow cell). **(b)** Representative kymographs of the full length of the DNA showing every second frame acquired (red: ORC^{JF646}; blue, $H2A^{AF488}$; note that DNA molecules can be captured in opposite orientations). The four conditions represented are DNA molecules containing (i) a non-chromatinized ARS1 origin, (ii) a chromatinized ARS1 origin, (iii) a non-chromatinized mutated origin, and (iv) a chromatinized mutated origin**.** Created with BioRender.com.

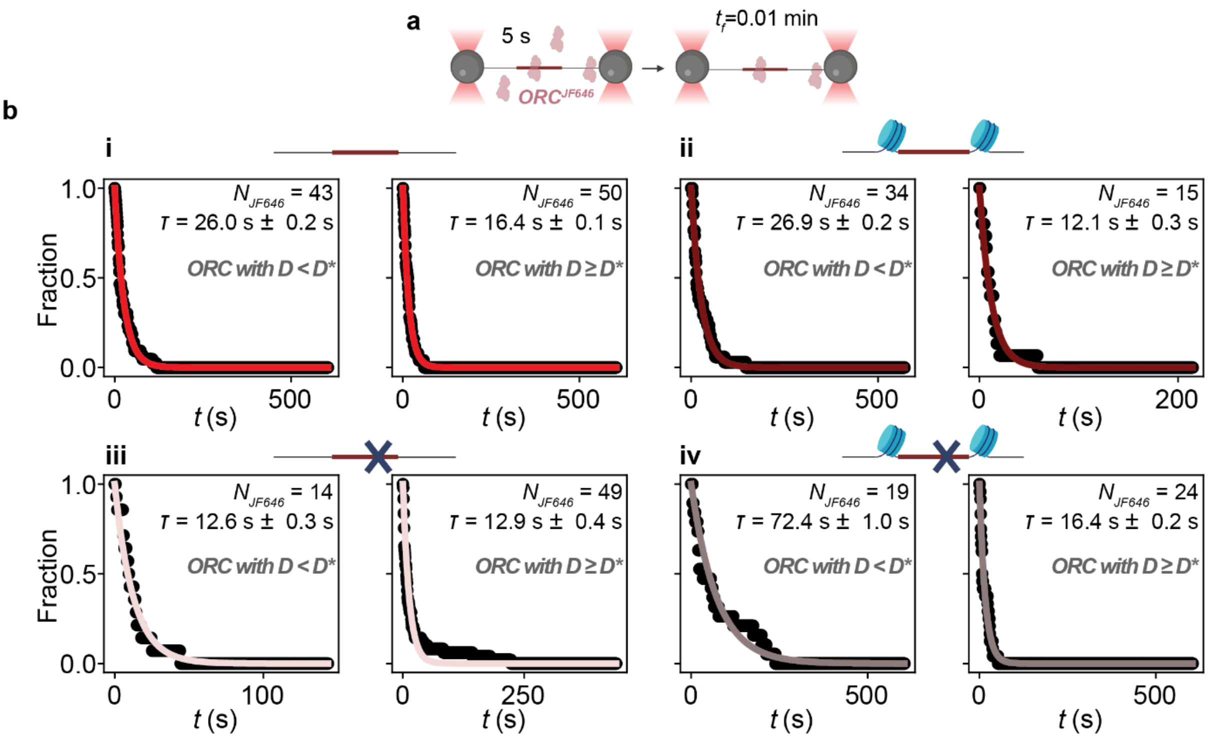

Supplementary Figure 3.2. Single-molecule lifetime analysis of rapidly bound ORC^{JF646} on 10.4 kbp DNA containing an ARS1 (or mutated) origin (chromatinized or not). (a) Tethered DNA molecules (chromatinized or not, as indicated) are introduced into the protein channel (Ch4) for incubation with ORC^{JF646} and Cdc6 for 5 s in the presence of ATP, and then moved into the buffer channel (Ch3) for confocal scanning at an acquisition frequency of one frame every 0.01 min (t_f = 0.01 min) (see **Figure 1b** for schematic of the flow cell). **(b)** (i) (left panel) Survival probability of single JF646 dyes (N_{JF646} = 43) associated to slow ORC^{JF646} on DNA containing an origin as deduced from the diffraction-limited red spots with $D < D^*$. (right panel) Survival probability of single JF646 dyes (N_{JF646} = 50) associated to fast ORC^{JF646} on DNA containing an origin as deduced from the diffraction-limited red spots with $D \geq D^*$. (ii) Survival probability of single JF646 dyes (N_{JF646} = 34) associated to slow ORC^{JF646} on DNA containing an origin as deduced from the diffraction-limited red spots with $D < D^*$. (right panel) Survival probability of single JF646 dyes (N_{JF646} = 15) associated to fast ORC^{JF646} on DNA containing an

origin as deduced from the diffraction-limited red spots with $D \geq D^*$. (iii) (lower left panel) Survival probability of single JF646 dyes (N_{JF646} = 14) associated to slow ORC^{JF646} on DNA containing an origin as deduced from the diffraction-limited red spots $D < D^*$. (right panel) Survival probability of single JF646 dyes (N_{JF646} = 49) associated to fast ORC^{JF646} on DNA containing an origin as deduced from the diffraction-limited red spots with $D \geq D^*$. (iv) (lower left panel) Survival probability of single JF646 dyes (N_{JF646} = 19) associated to slow ORC^{JF646} on DNA containing an origin as deduced from the diffraction-limited red spots with $D < D^*$. (right panel) Survival probability of single JF646 dyes (N_{JF646} = 24) associated to fast ORC^{JF646} on DNA containing an origin as deduced from the diffraction-limited red spots with $D \geq D$. Data are presented as mean values ± the standard deviation, computed from the covariance of the optimal parameters found by the least squares fit. Created with BioRender.com.

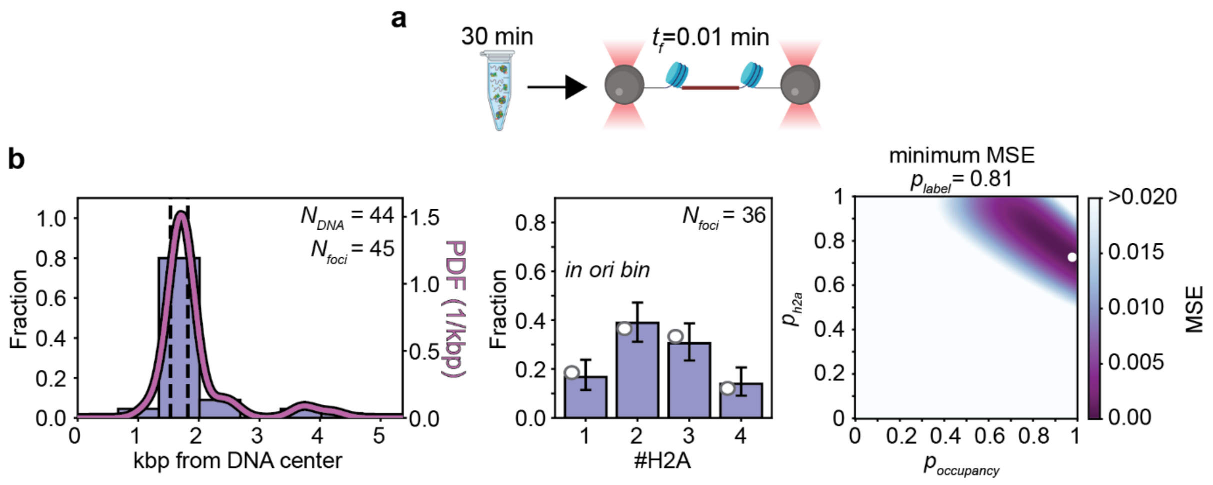

Supplementary Figure 4.1. Stability of a labeled chromatinized ARS1 origin on 10.4 kbp DNA. (a) DNA molecules containing a chromatinized ARS1 origin are incubated in bulk for 30 min in the presence of ATP. Subsequently, the DNA-protein construct is flushed into the single-molecule flow cell, tethered, and into Ch3 for confocal scanning at an acquisition frequency of one frame every 0.01 min (t_f = 0.01 min) (see **Figure 1b** for schematic of the flow cell). Created with BioRender.com. **(b)** (left panel) Spatial distribution of H2A^{AF488} on DNA as described in **Figure 1b**, acquired immediately after introduction into the flow cell and deduced from the blue diffraction-limited spots (N_{foci}) collected from a number of DNA molecules indicated by N_{DNA}. Dashed lines indicate the location of the NPS sites, and the solid curve indicates the kernel density estimation of the data (PDF: probability density function). (center panel) Stoichiometry distribution of H2A^{AF488} in the bin containing the chromatinized origin. Data are presented as mean values ± one sigma Wilson confidence intervals. Filled white circles indicated at left designate the fitted values based on the model described in the Methods and in **Supplementary Figure 1.3**. (right panel) Landscape of the mean squared error (MSE) between fit and experiment, calculated for different values of p_{h2a} and $p_{occupancy}$ at a fixed value of p_{label} = 0.81. The white dot indicates the optimal solution with the lowest error. Data derives from one chromatinized sample. Source data are provided as a Source Data file.

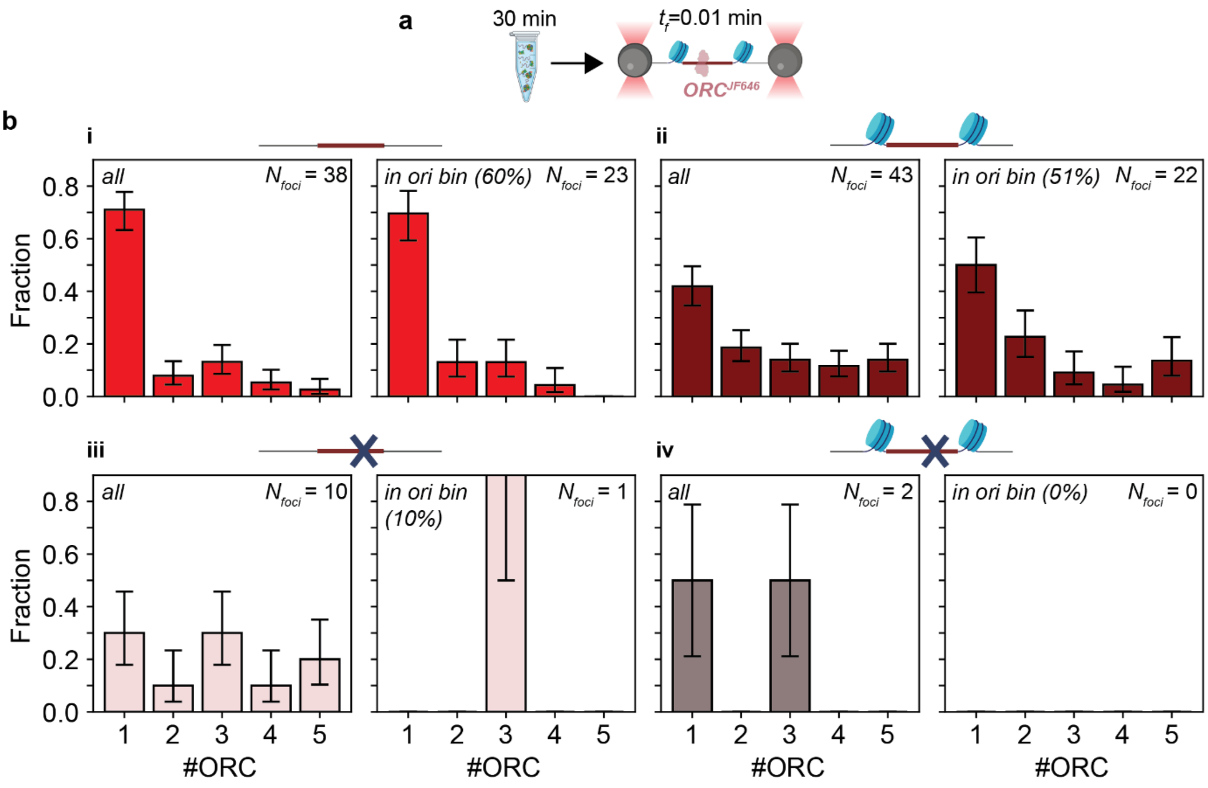

Supplementary Figure 4.2. Stoichiometry of stably bound ORC^{JF646} on 10.4 kbp DNA containing an ARS1 (or mutated) origin (chromatinized or not). (a) ORC^{JF646} and Cdc6 are incubated with DNA molecules (chromatinized or not, as indicated) in bulk for 30 min in the presence of ATP. Subsequently, the DNA-protein construct is flushed into the single-molecule flow cell, tethered, and moved into Ch3 for confocal scanning at an acquisition frequency of one frame every 0.01 min (t_f = 0.01 min) (see also **Figure 4**).**(b)** Stoichiometry of ORC^{JF646}foci (N_{foci}) pertaining to data shown in **Figure 4**. (left panels) Stoichiometry distributions of ORC^{JF646} over the entire DNA molecule and (right panels) stoichiometry distribution of ORC^{JF646} in the bin containing (i) the non-chromatinized ARS1 origin; (ii) the chromatinized ARS1 origin; (iii) the non-chromatinized mutated origin; and (iv) the chromatinized mutated origin, as also indicated by the schematics. Data are presented as mean values ± one-sigma Wilson confidence intervals. Created with BioRender.com.

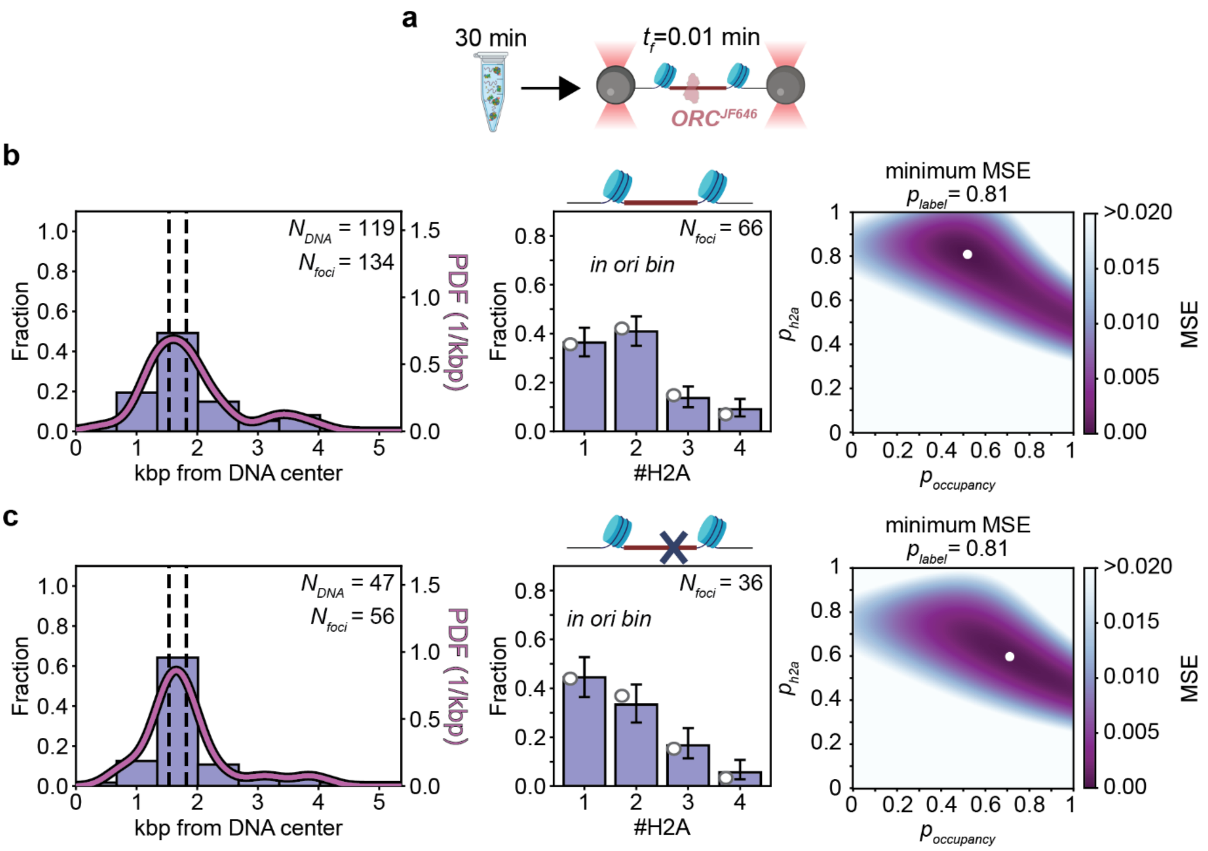

Supplementary Figure 4.3. Stability of a labeled chromatinized ARS1 (or mutated) origin on 10.4 kbp DNA in the presence of ORC^{JF646} and Cdc6. (a) ORC^{JF646} and Cdc6 are incubated with DNA molecules (chromatinized or not, as indicated) in bulk for 30 min in the presence of ATP. Subsequently, the DNA-protein construct is flushed into the single-molecule flow cell, tethered, and moved into Ch3 for confocal scanning at an acquisition frequency of one frame every 0.01 min (t_f = 0.01 min) (see **Figure 1b** for schematic of the flow cell). **(b)** (left panel) Spatial distribution of H2A^{AF488} on DNA containing a chromatinized ARS1 origin as described in **Figure 1b**, acquired immediately after introduction into the flow cell and deduced from the blue diffraction-limited spots (N_{foci}) collected from a number of DNA molecules indicated by N_{DNA}. Dashed lines indicate the location of the NPS sites, and the solid curve indicates the kernel density estimation of the data (PDF: probability density function). (center panel) Stoichiometry distribution of H2A^{AF488} in the bin containing the chromatinized origin. Data are

presented as mean values ± one-sigma Wilson confidence intervals. Filled white circles indicated at left designate the fitted values based on the model described in the Methods and in **Supplementary Figure 1.3**. (right panel) Landscape of the mean squared error (MSE) between fit and experiment, calculated for different values of p_{h2a} and $p_{occupancy}$ at a fixed value of p_{label} = 0.81. The white dot indicates the optimal solution with the lowest error. Data derives from one chromatinized sample. **(c)** (left panel) Spatial distribution of $H2A^{AF488}$ on DNA containing a chromatinized mutated origin, determined as described in panel (b). (center panel) Stoichiometry distribution of $H2A^{AF488}$ in the bin containing the chromatinized mutated origin. (right panel) Landscape of the mean squared error (MSE) between fit and experiment, calculated as in panel (b). Data derives from one chromatinized sample. Source data are provided as a Source Data file. Created with BioRender.com.

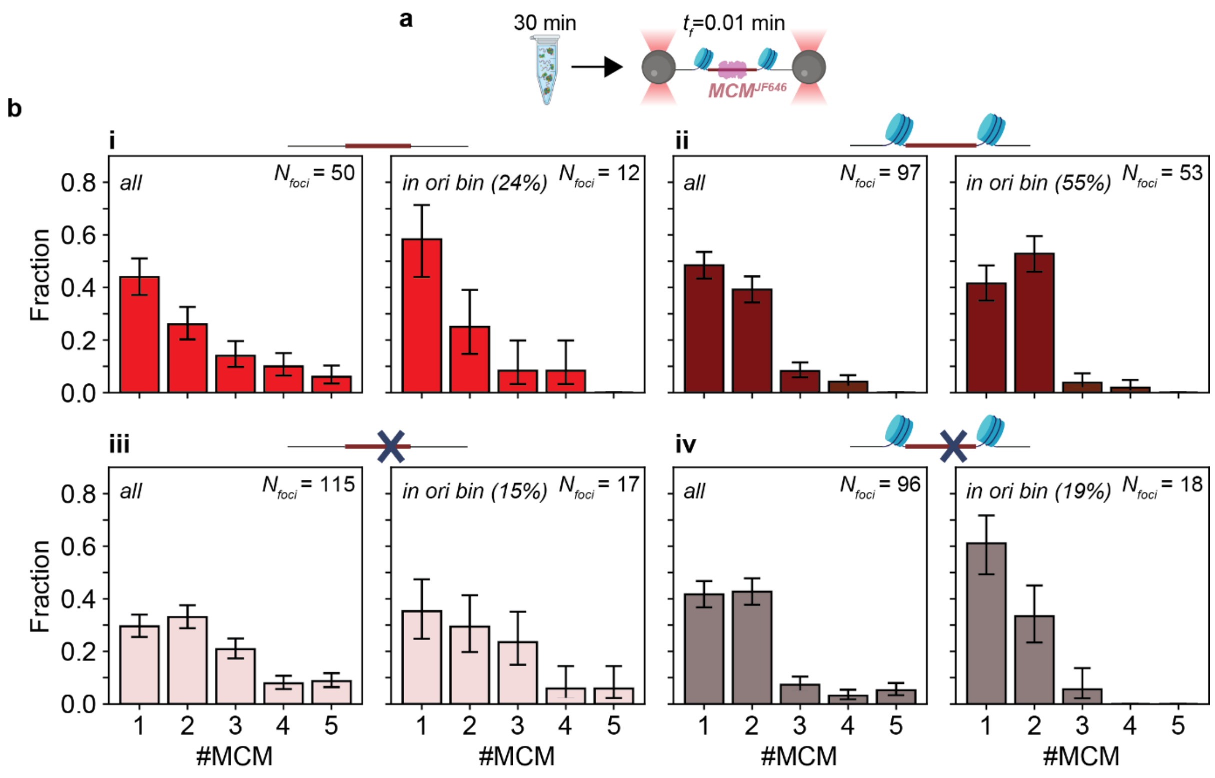

Supplementary Figure 5.1. Stoichiometry of MCM^{JF646} on 10.4 kbp DNA containing an ARS1 (or mutated) origin (chromatinized or not). (a) ORC, Cdc6, and MCM^{JF646}/Cdt-1 are incubated with 10.4 kb DNA molecules (chromatinized or not, as indicated) in bulk for 30 min in the presence of ATP. Subsequently, the DNA-protein construct is flushed into the single-molecule flow cell, tethered, and moved into into Ch3 for confocal scanning at an acquisition frequency of one frame every 0.01 min (t_f = 0.01 min) (see also **Figure 5**). **(b)** Stoichiometry of MCM^{JF646} foci (N_{foci}) from data shown in **Figure 5.** (left panels) Stoichiometry distributions of MCM^{JF646} over the entire DNA molecule and (right panels) stoichiometry distribution of MCM^{JF646} in the bin containing (i) the non-chromatinized ARS1 origin; (ii) the chromatinized ARS1 origin; (iii) the non-chromatinized mutated origin; and (iv) the chromatinized mutated origin, as also indicated by the schematics. Data are presented as mean values ± one-sigma Wilson confidence intervals. Created with BioRender.com.

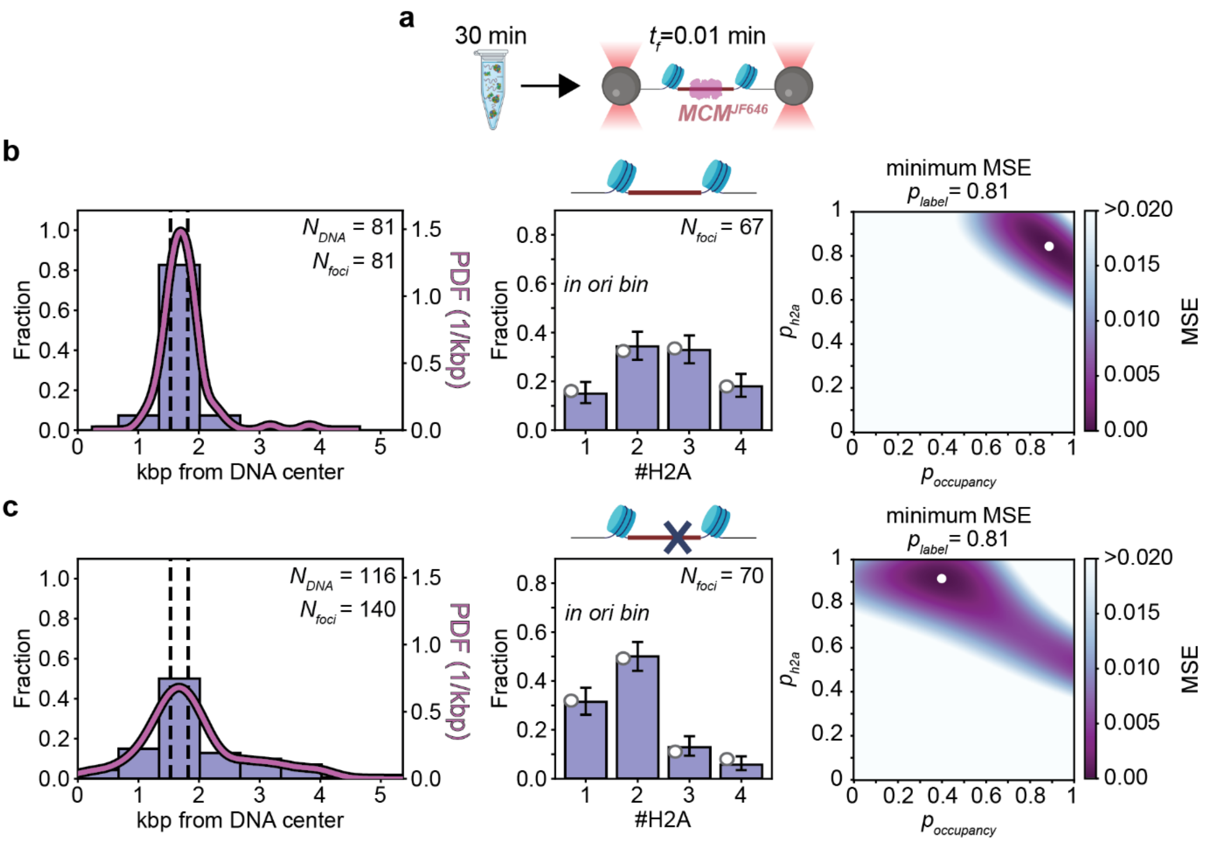

Supplementary Figure 5.2. Stability of a labeled chromatinized ARS1 (or mutated) origin on 10.4 kbp DNA in the presence of MCM^{JF646}, ORC, and Cdc6. (a) ORC, Cdc6, and MCM^{JF646}/Cdt1 /Cdt-1 are incubated with 10.4 kb DNA molecules (chromatinized or not, as indicated) in bulk for 30 min in the presence of ATP. Subsequently, the DNA-protein construct is flushed into the single-molecule flow cell, tethered, and moved into into Ch3 for confocal scanning at an acquisition frequency of one frame every 0.01 min (t_f = 0.01 min) (see **Figure 1b** for schematic of the flow cell). **(b)** (left panel) Spatial distribution of H2A^{AF488} on DNA containing a chromatinized ARS1 origin as described in **Figure 1b**, acquired immediately after introduction into the flow cell and deduced from the blue diffraction-limited spots (N_{foci}) collected from a number of DNA molecules indicated by N_{DNA}. Dashed lines indicate the location of the NPS sites, and the solid curve indicates the kernel density estimation of the data (PDF: probability density function). (center panel) Stoichiometry distribution of H2A^{AF488} in the bin containing

the chromatinized origin. Data are presented as mean values ± one-sigma Wilson confidence intervals one-sigma Wilson confidence intervals. Filled white circles indicated at left designate the fitted values based on the model described in the Methods and in **Supplementary Figure 1.3**. (right panel) Landscape of the mean squared error (MSE) between fit and experiment, calculated for different values of p_{h2a} and $p_{occupancy}$ at a fixed value of p_{label} = 0.81. The white dot indicates the optimal solution with the lowest error. Data derived from two chromatinized samples. **(c)** (left panel) Spatial distribution of $H2A^{AF488}$ on DNA containing a chromatinized mutated origin, determined as described in panel (b). (center panel) Stoichiometry distribution of $H2A^{AF488}$ in the bin containing the chromatinized mutated origin. (right panel) Landscape of the mean squared error (MSE) between fit and experiment, calculated as in panel (b). Data derived from two chromatinized samples. Source data are provided as a Source Data file. Created with BioRender.com.

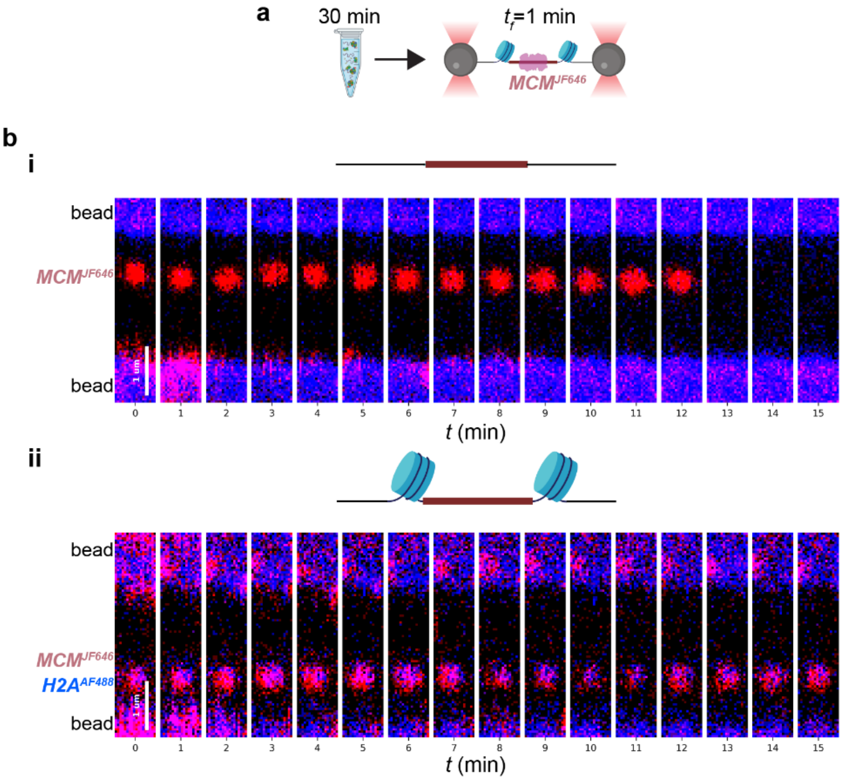

Supplementary Figure 5.3. Representative kymographs of MCM^{JF646} on 10.4 kbp DNA containing an ARS1 origin (chromatinized or not). (a) ORC, Cdc6, and MCM^{JF646}/Cdt1 are incubated with 10.4 kb chromatinized DNA molecules in bulk for 30 min in the presence of ATP. Subsequently, the DNA-protein construct is flushed into the single-molecule flow cell, tethered, and moved into Ch3 for confocal scanning at an acquisition frequency of one frame every 0.01 min (t_f = 0.01 min) (see **Figure 1b** for schematic of the flow cell). **(b)** Representative kymographs of the full length of the DNA showing every frame acquired (red: MCM^{JF646}; blue, H2A^{AF488}). The two conditions represented are for DNA molecules containing (i) a non-chromatinized ARS1 origin, and (ii) a chromatinized ARS1 origin**.** Created with BioRender.com.

Supplementary Tables

Supplementary Table 1. Names and sequences of all primers used in this study.

Primer number	sequence
TL-001	ATACCTCTATACTTTAACGTCAAGGAG
TL-002	AGCGAGATGATAATCCTGTGAGGG
TL-019	CAAGGCTGTGGACATCGGCCCAGGTCTGAATCTGCTGC
TL-020	GCAGCAGATTCAGACCTGGGCCGATGTCCACAGCCTTG
TL-021	TGTTAGTTAGTTACTTAAGCTCG
TL-022	TCGAGTGCGGCCGCGAATTCG
TL-023	GCCCGCGAGACCTTCCAGGCATTCCGCACCACCGACG
TL-024	CGTCGGTGGTGCGGAATGCCTGGAAGGTCTCGCGGGC
TL-025	TCCCCCCGGGGGGAATGGCAGAAATCGGTACTGG
TL-026	TCCCCCCGGGGGGAAGCCGGAAATCTCGAGCGTCG
TL-027	TAAAATGTTGCTGAGGTGAGG
TL-028	TAGCAGCAGAAACAGCAATGAAG
TL-063	ATTAGTTTTTTAGCCTTATTTCTGG
TL-064	TATGATTATTAAACTTCTTTGCG
TL-084	ATGGCAGAAATCGGTACTGG
TL-087	CGACGCTCGAGATTTCCGGCGGTGAAATGGCTAAGACTTTGAAG
TL-119	TATTAAACTTCTTTGCGTCCATCC
TL-441	AAGAAGCTAAGAGAGCTATGAACGAAGACGAAAC
TL-443	TAAGGCGCGCCTATAAAACAATGGCTAAGACTTTGAAGGACTTGC
TL-446	TAAGGCGCGCCTATAAAACAATGAAGAGAAGATGGAAGAAGAACTTCAT

TL-447	GATTGGTTCAAGTCAGACATGCCGGAAATCTCGAGC
TL-449	TAACCCTCACTAAAGGGAACAAAAGC
TL-470	AAGTTGTACAGAGAAGCTAAC
TL-472	CGACGCTCGAGATTTCCGGCATGTCTGACTTGAACCAATCTAAGAAGATGAACG
TL-473	TTCTTCCATCTTCTCTTCATTGTTTTATAGGCGCGCCTTATATTGAATTTTC
TL-817	GGCGCGCCCCGCCCTGGAGAATCGCGGTG
TL-818	GGCGCGCCCCTGCACCCCAGGGACTTG
TL-959	ATCGAGGAATTCGATGTATATATCTGACACGTGCCTGGAG
TL-960	GATATATACATCGAATTCCTCGATTTTTTTATGTTTAGTTTCGCG
TL-961	GCGCGAACGGCCAGCCTGGAACCG
TL-962	GCTGGCCGTTCGCGCGCCCAC
TL-963	TGACCATGATTACGCCACCTGCACCCCAGGGAC
TL-964	GATATATACATCGAATTCCTCGATTTTTGGATGGGGAG
HS_BN23NPb	GGTCTCGCCCTCCGCCCTGGAGAATCGC
HS_BN26NPb	GGTCTCGGCACCTGCACCCCAGGGAC
HS_BN45	GGAGGGTCTCCGTGCAAG
HS_BN46	Bio-Bio-Bio-GGTTCCCTACTCTCGCATGC
HS_BN47	Bio-Bio-Bio-CTCGAGTGCCAGGCATCAAAG
HS_BN48	GGGGTCTCCAGGGAGAAATTACAC

Supplementary Table 2. Names and sequences of all gBlocks^{TM} gene fragments used in this study.

gBlocks^{TM} gene fragments	Sequence
pTL013	CCGCCCTGGAGAATCGCGGTGCCGAGGCCGCTCAATTGGTCGTAGACAGCTCTA<br>GCACCGCTTAAACGCACGTACGCGCTGTCCCCCGCGTTTTAACCGCCAAGGGGAT<br>TACTCCCTAGTCTCCAGGCACGTGTCAGATATATACATCCTGTATTTTACAGATTTT<br>ATGTTTAGATCTTTTATGCTTGCTTTTCAAAAGGCCTGCAGGCAAGTGCACAAACA<br>ATACTTAAATAAATACTACTCAGTAATAACCTATTTCTTAGCATTTTTGACGAAATT<br>TGCTATTTTCCCAGTTCGCGCGCCCACCTACCGTGTGAAGTCGTCACTCGGGCTTC<br>TAAGTACGCTTAGCGCACGGTAGAGCGCAATCCAAGGCTAACCACCGTGCATCG<br>ATGTTGAAAGAGGCCCTCCGTCCTTATTACTTCAAGTCCCTGGGGTGCAGG
pGC218	GAATTCCTCGATTTTTGGATGGGGAGTTTCGCGGACGACGGTTTCGAGGTGGCG<br>GTCTGGACCACGCCGGAGAGCGTCGAAGCGGAGGCGGTGTTCGCCGAGATCGG<br>CTCGCGCATGGCCGAGTTGAGCGGTTCCAGGCTGGCCAAACAGCATCAGATGGA<br>ACTCCCCATACCAATACACCGGCCTCAGCATCCGGTACCTCAGCTGGCCACCGTCG<br>GCGTCTCGCACGACCACCAGTGCAAGGGTCTGAGCAGCGCCGTCGTGCTCCTCG<br>GAGTGGAGGCAGCCGAGCGCGACGGTGTGCCCGCCTTCCTGGAGACCTCCGCGC<br>TCCGCAACCTCCACTTCTACGAGCGGCTCGGCTTCACCGTCACCGCCGACGTCGA<br>GGTGCCCGAAGGACCGCGCACCTGGTGCATGACCCGCAAGCCCGGTGCCTGACG<br>CTCGCCACACGACCCGCAGCGCCCGACCGAAAGGAGCGCACGACCCGGG
